# Covalent Organic Frameworks: From Structures to Applications

**DOI:** 10.3390/polym15051279

**Published:** 2023-03-02

**Authors:** Quang Nhat Tran, Hyun Jong Lee, Ngo Tran

**Affiliations:** 1 Department of Chemical and Biological Engineering, Gachon University, 1342 Seongnamdaero, Sujeong-gu, Seongnam-si 13120, Republic of Korea; 2 Institute of Research and Development, Duy Tan University, Da Nang 550000, Vietnam; 3 Faculty of Natural Sciences, Duy Tan University, Da Nang 550000, Vietnam

**Keywords:** covalent organic frameworks, different topologies, applications

## Abstract

Three-dimensional covalent organic frameworks possess hierarchical nanopores, enormous surface areas with high porosity, and open positions. The synthesis of large crystals of three-dimensional covalent organic frameworks is a challenge, since different structures are generated during the synthesis. Presently, their synthesis with new topologies for promising applications has been developed by the use of building units with varied geometries. Covalent organic frameworks have multiple applications: chemical sensing, fabrication of electronic devices, heterogeneous catalysts, etc. We have presented the techniques for the synthesis of three-dimensional covalent organic frameworks, their properties, and their potential applications in this review.

## 1. Introduction

Covalent organic frameworks (COFs) are two- or three-dimensional (2D, 3D) families of materials, which, through interactions between organic precursors, produce strong, covalent networks that enable the formation of porous, constant, and crystalline solids [1,2]. COFs are mainly generated as polycrystalline powders which limit the processing steps [3,4]. The powder form hinders its application in electrochemical processes as electrodes or separators [5], separation using membranes [6], size fractionation [7], and elimination of adsorbents [8]. The performances of electronic devices depend on the quality of active layers of films, which are affected by the COF’s insolubility during film fabrication. 

Functional groups are incorporated during the synthesis of COFs. These functional groups can be incorporated through two different methods: 2D layered structures [2,3] and 3D extended networks [4]. The incorporation strategy of functional groups is dictated by connecting points and the geometry of precursors. In addition, 2D flat sheets stack in a face-to-face approach. The functional groups present in the adjacent layers show a strong p–p interaction, which facilitates the movement of charges. 

The stacked layers are held together through noncovalent interactions, which impart a large intrinsic freedom to the layers. Two orthogonal phase spaces are used for classifying this freedom: the interlayer distance and the layer offset. The presence of a zero offset vector indicates the formation of eclipsed stacking (AA) between the consecutive layers. The presence of a nominal value of non-zero offset indicates slipped AA stacking. The slipped AA stacking forms a type of serrated stacking called AB stacking. The vertices of the layer lie above the pore centers of the consecutive layer to form the AB stacking.

On the other hand, 3D COFs form a framework with open sites and are bonded through strong covalent bonds. The functional groups in 3D COFs and 2D COFs align differently, resulting in different interaction patterns [8,9,10]. Thus, the same functional groups can be utilized to fabricate 2D and 3D COFs for investigating their properties.

Metal–organic frameworks (MOFs), 3D porous coordination polymers, are formed by secondary building blocks, such as organic ligands and metal ions and clusters. COFs and MOFs are both porous coordination polymers, which make them attractive candidates for various applications. They have porous volumes and π-conjugated bonds. An accurate choice of linkers can impart tunable properties. The bonds are concurrently formed and broken with organic linkers during the formation of a MOF structure which helps in the growth of good crystals. The secondary building blocks in MOFs can alter their structure and size, keeping the topology intact. The presence of long organic linkers makes them highly porous. In contrast, covalent bonds are formed rigorously in COFs, leading to the formation of poor crystalline structures [10,11]. Thus, COFs require a planned synthetic strategy to achieve their crystalline form. However, they are highly stable above 300 °C and are not easily soluble in solvents, as compared to MOFs.

The substitution of rigid building units with flexible building units imparts flexibility with significant characteristics in the presence of external stimuli [11]. Nevertheless, the presence of different conformations and crystallographic defects have hindered the fabrication of 3D COFs using flexible building blocks, which deters the exploitation of the dynamic characteristics of COFs [12].

It has been a challenge for the synthesis of 3D COFs to integrate with flexible building units, due to the absence of additional weak interactions between the interlayers [13]. The flexible building blocks have single bonds and the freedom of rotation is high around a single bond; this enhances the defects during the crystalline growth, encumbering the formation of 3D COF crystals [14]. Upon activation, 3D COFs have a tendency to collapse and lose crystallinity, creating a challenge to the analysis of the structure [15].

**Figure 1:**
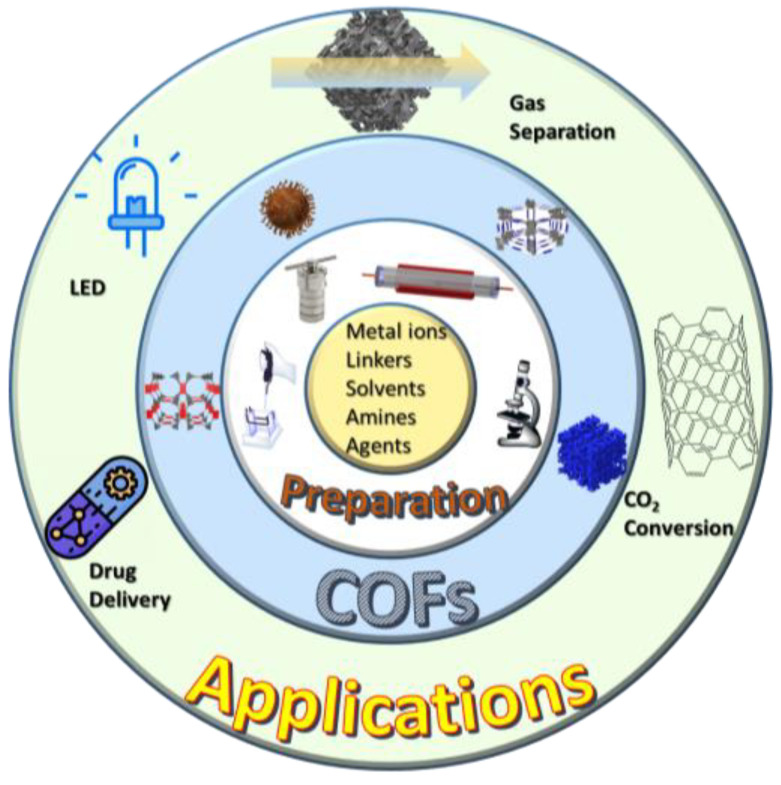
The overview of the prepare approaches of COFs and their applications.

Although there are few reviews on the application and project of 3D COFs [9,16] and the design of COFs [17,18], as shown in Figure 1, there are no new reviews exclusively on detailed and potential applications of 3D COFs. In this situation, this review elaborately discusses the comparative study of the different topologies and the application of 3D COFs. We reflected to bridge the gap by discussing the research works on COFs for the last few years.

## 2. Triagonal Prism Units

### 2.1. The stp Topology

**Figure 2:**
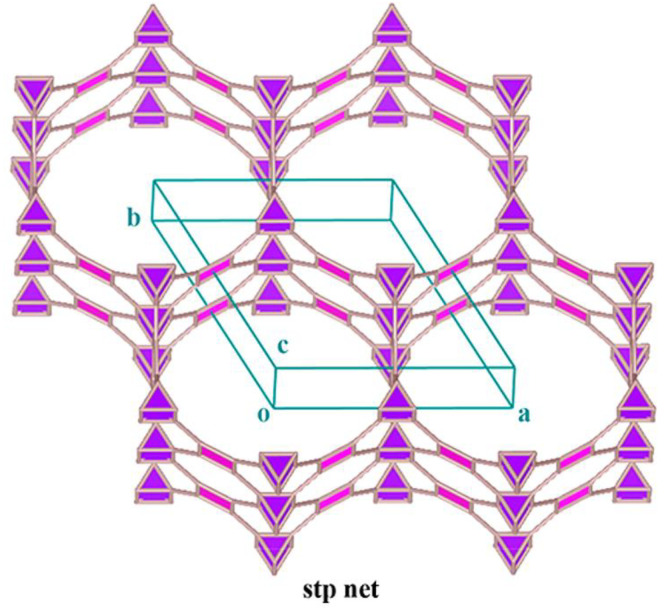
Stp topology for 3D COF-triptycene. Reprinted (adapted) with permission from [19]. Copyright {2020} American Chemical Society.

2,3,6,7,14,15-hexakis(4′-formylphenyl)triptycene (HFPTP), a building unit with six connections, was used to design a 3D COF (3D COF-triptycene) [19]. The crystal had an **stp** topology, low density, large pores, and a high surface area. The presence of wide channels within the crystal aided in the uptake of a large protein molecule, myoglobin, as shown in Figure 2.

**Scheme 1:**
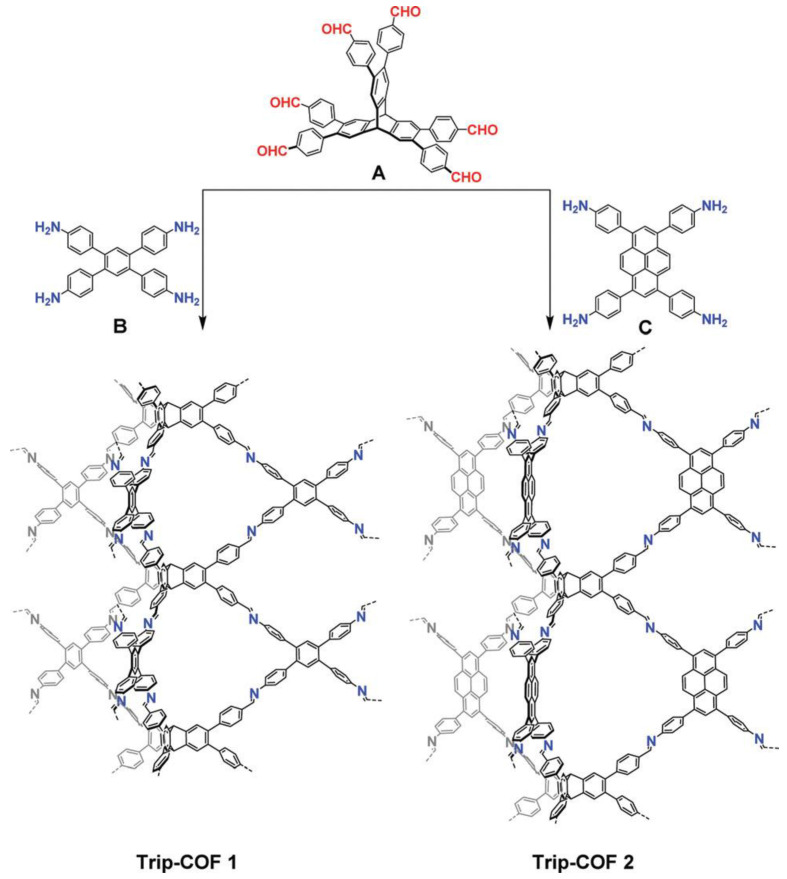
Synthesis of 3D-COF-trip-1 and 3D-COF-trip-2 [20].

Two triptycene-based COFs (3D COF-trip-1, 3D COF-trip-2) were synthesized by [6 + 4] a condensation reaction of imine, as shown in Scheme 1, with an **stp** topology [20]. Nanochannels with honeycomb geometry were present within the crystals. The pore sizes varied depending on the type of linkers. Both the crystals had 1D channels. Although hexagonal pores were present in 3D COF-trip-1, these pores were absent in 3D COF-trip-2 and were replaced by abundant 1D channels. Additionally, a second interpenetration took place in 3D COF-trip-2 to form excess space between the nearby units of triptycene. 

### 2.2. The tbo Topology

**Figure 3:**
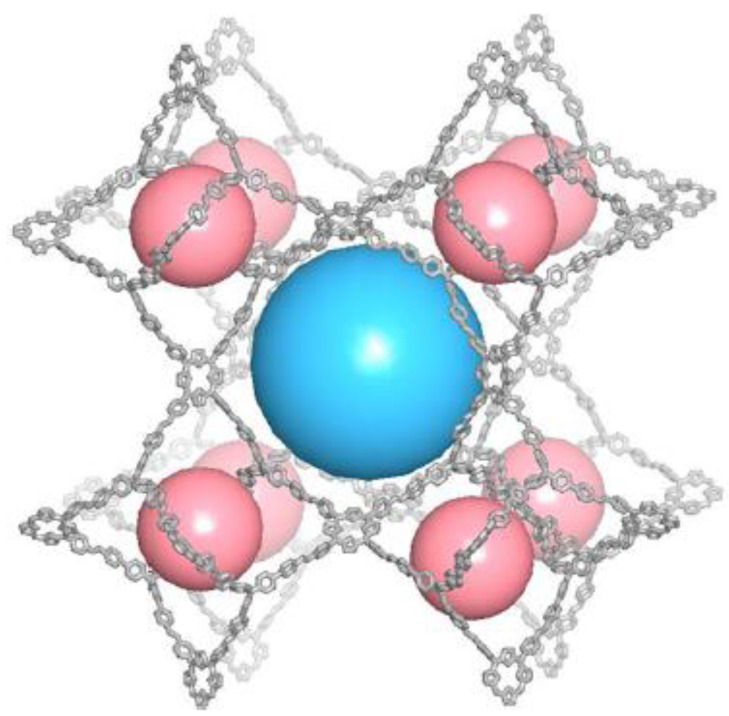
A 3D COF with tbo topology. Reprinted (adapted) with permission from [21]. Copyright {2020} American Chemical Society.

4′,4‴,4‴″-nitrilotris[(1,1′-biphenyl)-4-carbaldehyde] (NBC), a trigonal aldehyde with 3 connections, and 5,10,15,20-tetra(4-aminophenyl)porphyrin [H_2_(TAP)], an amine with 4 connections, were allowed to undergo [3 + 4] condensation to form a 3D COF with a **tbo** topology having a non-interpenetrated porous skeleton, as shown in Figure 3 [21]. The three square blocks were attached to a tritopic bridging unit to form a *T_d_*-octahedron. The linkers were placed on the triangular faces of the truncated octahedron alternately. There were 3 types of polyhedral cages in this topology. The COF is a good photocatalyst for the removal of fluorine from trifluoromethyl aromatics and the conversion of phenylboronic acids to phenols. The photocatalytic reaction took place both on the surface of the COF and within the crystal.

### 2.3. The ceq Topology

**Figure 4:**
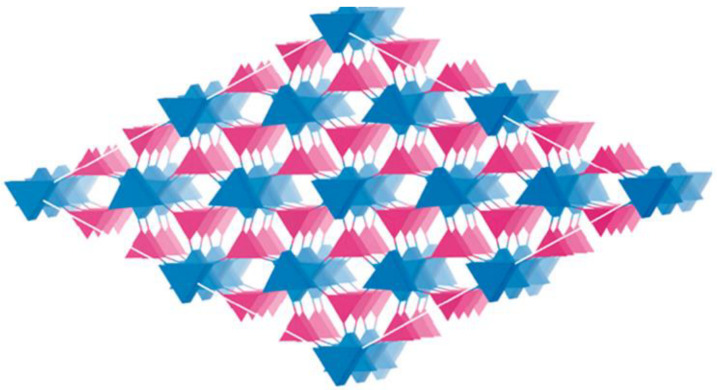
The structure of 3D COF-trip with ceq net. Reprinted (adapted) with permission from [22]. Copyright {2021} American Chemical Society.

The vertex of a stereo triangular prism and a triangular linker were combined with a **ceq** 6,3-connected topology using triptycene to form a 3D COF with a two-fold interpenetrated geometry [22], as shown in Figure 4. The crystal is porous and has an ordered geometry. The crystal adsorbed carbon dioxide selectively from a gas mixture due to the interaction of the aromatic rings with the gas. Additionally, the imine groups which have an excess of electrons also combine with carbon dioxide and add to the adsorption activity. 

## 3. Topology of 3D COFs Using Octahedral Units

### The pcu Topology

**Figure 5:**
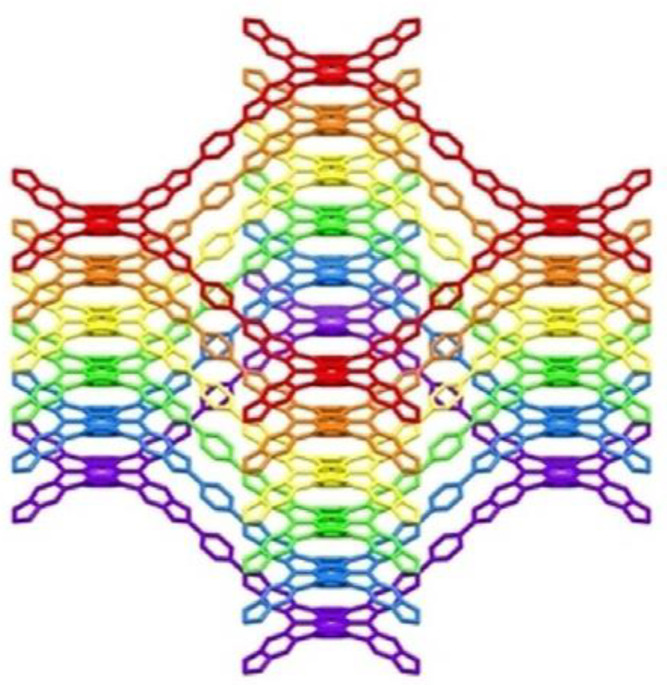
The structure 3D COF-HBC with pcu topology [23].

2,3,6,7,10,11,14,15,18,19,22,23-dodecahydroxy-*cata*-hexabenzocoronene (HBC), a compound with a *D_3d_* antiprismatic triangular geometry, was employed as a monomer for the synthesis of 3D COFs [23]. The monomer is a polycyclic aromatic compound with a distorted geometry. The monomers are condensed with linear diboronic acids to form crystals (3D COF-HBC) with a topology of **pcu** net with six-fold interpenetration, as shown in Figure 5. The triangle-shaped micropores, formed by the trigonal trapezohedral lattice, combine to form wide channels. The π−π stacking between the nodes of the framework combines the π-interpenetrated structure. The self-assembly of *cata*-hexabenzocoronene favors the π-interpenetrated structure, which enhances the charge transport characteristics of the 3D COF. The nanocrystallites have the shape of a desert rose, where the petals move out from the center. Internal pores were revealed by the removal of the surface layer containing the flower-like structure. 

## 4. Topologies of Metalated 3D COFs

### 4.1. The pts Topology

**Figure 6:**
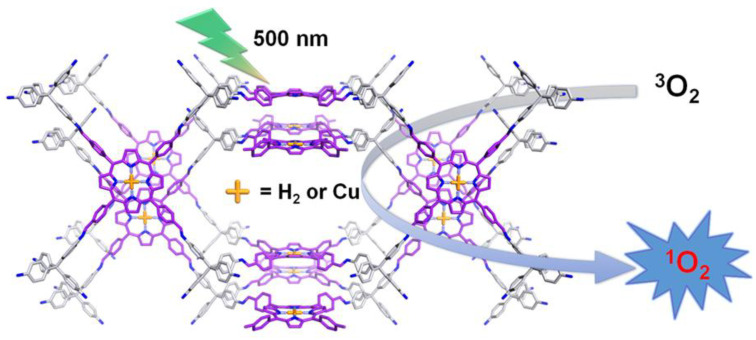
Crystal structure of 3D CuPor-COF. Reprinted (adapted) with permission from [24]. Copyright {2017} American Chemical Society.

Another novel COF containing Cu and porphyrin (3D CuPor-COF) was prepared using a [4 + 4] condensation reaction with square precursors (2D-C_4_), having a **pts** topology [24]. However, these crystals produced singlet oxygen weakly on photoirradiation, as shown in Figure 6.

### 4.2. The dia Topology

**Figure 7:**
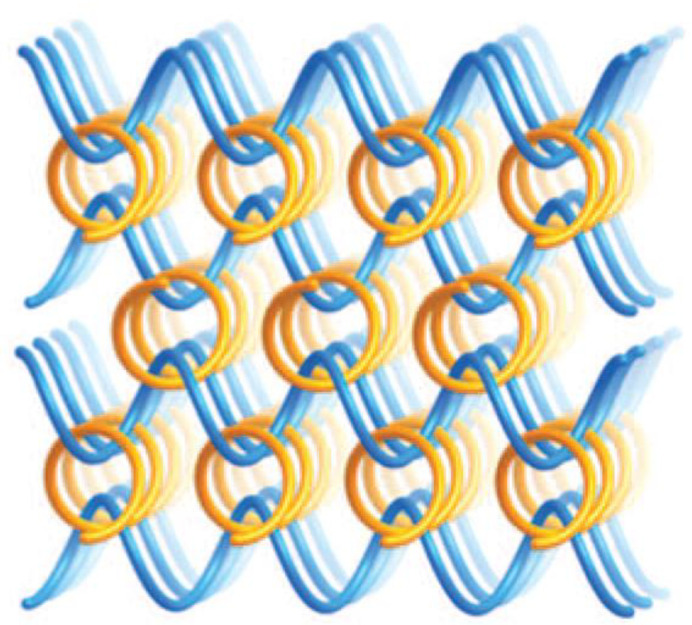
Schematic representation of weaving threads in three-dimension [25].

A metal-containing COF (COF-505) was woven using helical threads of organic molecules, which exhibited good elastic properties [25]. A 3D architecture was created by interlacing the 1D units, allowing extensive spatial deformation for each thread on the one hand, but retaining the topology on the other hand. Thus, the mechanical characteristics of the COF could be reversibly controlled by the allowed freedom. The tetrahedral geometry of Cu(I)-bis[4,4′-(1,10-phenanthroline-2,9-diyl) dibenzaldehyde] tetrafluoroborate [Cu(PDB)_2_(BF_4_)] was exploited by using it as a building block that was combined with benzidine to synthesize the imine threads, as shown in Figure 7. The pores were filled by tetrafluoroborate anions. Cu(I) systematically compiled the threads by behaving as templates, and the alignment of 1,10-phenanthroline-2,9-diyl dibenzaldehyde units proved that the threads were independently produced and not dependent topologically on the Cu(I) ions. The threads self-assembled into a 3D architecture propelled by the tetrahedral geometry of the aldehyde units following **dia** topology. The topology and architecture did not change after removing the metal ion. However, on remetalation with Cu(I), the complete structure was refurbished reversibly. The aldehyde groups were present in the para positions of both the phenyl substituents. A distorted tetrahedral geometry was formed where the two planes of phenanthroline form a dihedral angle of 57° with Cu(I) at the center. The π−π interaction between the neighboring planes of phenyl units and phenanthroline distorted the tetrahedral geometry [26]. The threads were covalently bonded and propagated in two opposite directions along [−110] and [110] to form chemically similar helices with opposite chirality, resulting in a racemic woven framework. The COF exhibited high elasticity on removing Cu(I) as the interaction between the threads became weak. 

A metal was incorporated into JUC-509 [27] and was explored for the elimination of the superoxide radical anion (O_2_^•−^), since salphens with metal centers are good antioxidant catalysts [28]. The presence of copper in the salen-based COF showed good antioxidant activity and the catalyst was devoid of any leaching of metal ions. The crystals were recovered and reused three times, keeping the activity intact. 

Zn metal was incorporated in the salen-based COFs and was used as a stationary phase of columns [29]. However, these metalated salen-based COFs were not effective at separating ethylenebenzene and xylene isomers. Since the polar salen groups remained coordinated with the Zn-metal, they could not interact with the methyl groups of xylene isomers and failed to separate them. Thus, the polar salen groups were inevitable for the recognition of the isomers. Moreover, the xylene isomers interacted with the salen-COF-Zn through the metal sites and possessed equal adsorption energies, which hindered the possible separation of the isomers. 

### 4.3. The bor Net Topology

**Figure 8:**
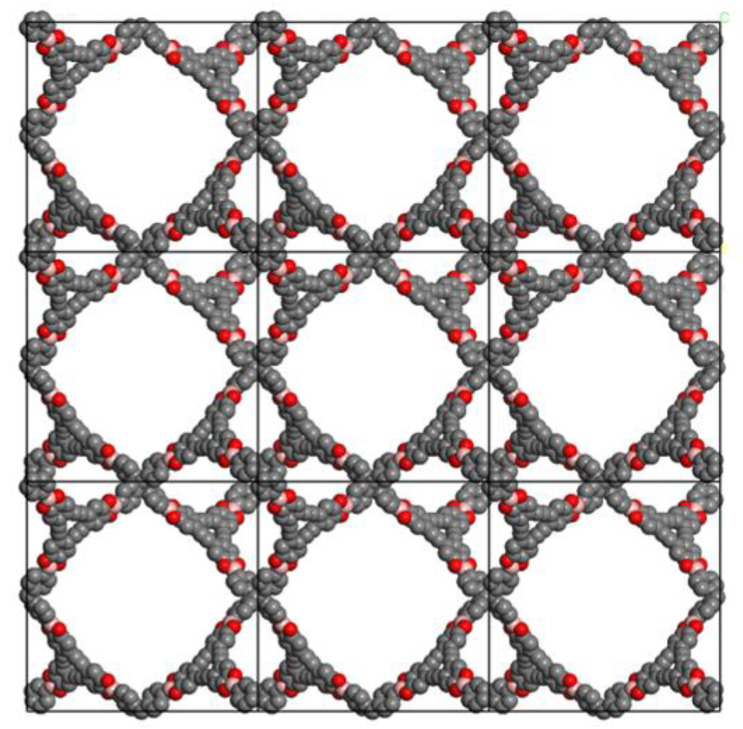
The schematic representation of the **bor** net topology. Reprinted (adapted) with permission from [30]. Copyright {2016} American Chemical Society.

The fabrication of metalated COFs without affecting the pore volume or surface area is not easily feasible. Thus, selecting a monomer with an accurate geometry for creating crystalline networks and forming stable complexes with metal ions has been a challenge for synthetic chemists. In one report [30], tetrahedral-shaped tetra-(4-dihydroxyborylphenyl)methane (TBPM) and dehydrobenzoannulene units containing C_3_-symmetric conjugated π-electrons were combined to create a mesoporous 3D COF (DBA-3D-COF), which was later metalated, as shown in Figure 8. Ni (10 wt%) was incorporated into the prepared COF using Ni(COD)_2_ to form Ni-DBA-3D-COF, which showed good absorption capacity for ethylene and ethane. The metal links to the dehydrobenzoannulene cavity, devoid of any interaction at the bond between boronate and ester, created a Ni(0) complex [31]. However, the luminescence characteristics of the metal-free COF were quenched after the incorporation of Ni. The rate of ethylene and ethane uptake was more for metalated COFs, compared to those of COFs without a metal. The factors responsible for the higher absorption rate are due to the following reasons: (1) a π-complex is formed between ethylene π orbitals and Ni(0) d-orbitals complex through a weak interaction [32] and (2) ethane is polarized by the open Ni(0) site [33]. However, the binding interactions between ethylene and ethane are not enhanced by the open metal sites.

### 4.4. The soc Topology

**Figure 9:**
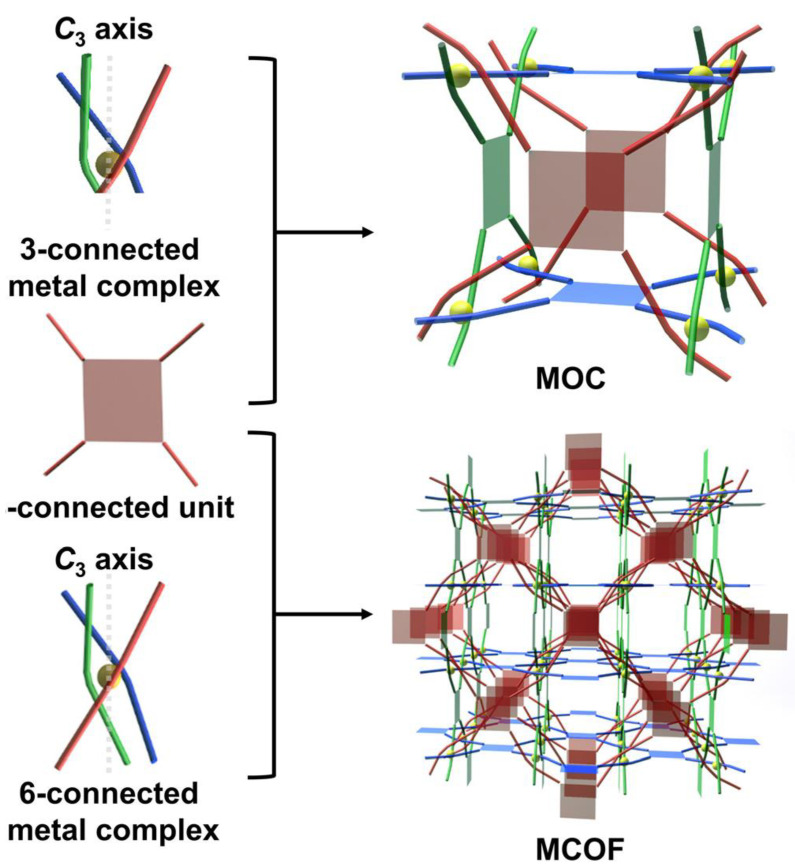
Conversion strategy of metal-organic cages to 3D COFs [34].

An anionic titanium-based 3D COF with **soc** topology was synthesized by the conversion of metal-organic cages to metal-based 3D COFs [34]. 4,4′,4′’,4‴-(pyrene-1, 3, 6, 8-tetrayl)tetraaniline (PyTTA), a four-connected building block, was condensed with Na_2_Ti(2,3-DHTA)_3_ having six-connected nodes through an imine linker. The anionic skeleton was stabilized by sodium as a cation. Meerwein addition was photocatalyzed by the 3D COF-Ti. The catalyst was recycled five times with little loss in its efficacy, as depicted in Figure 9.

### 4.5. The nbo Topology

**Figure 10:**
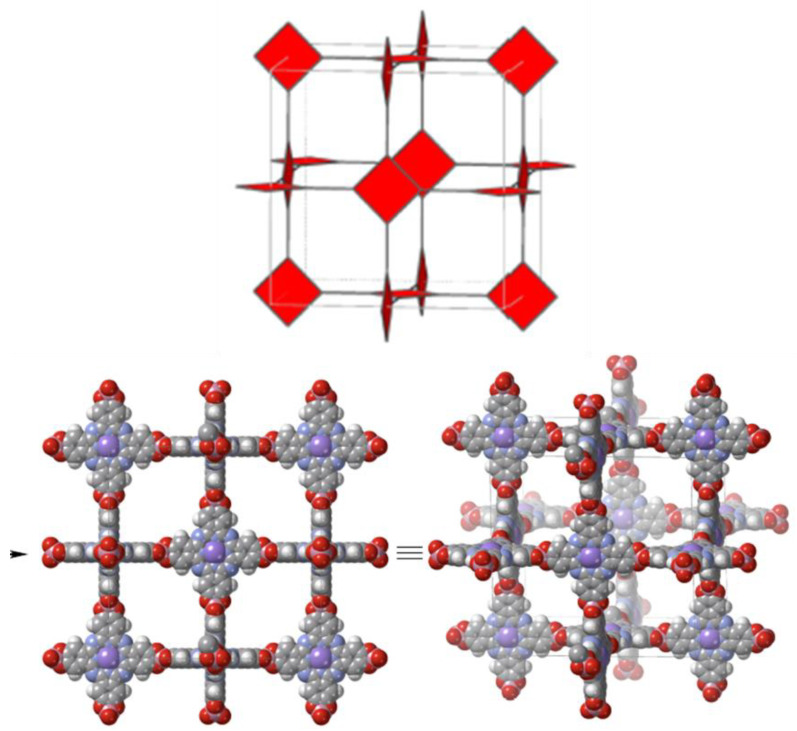
The schematic representation of the nbo topology [35].

Spiroborate linkers with negatively charged sp^3^ hybridized boron centers were used for the synthesis of a 3D COF (3D COF-SB) with **nbo** topology, as shown in Figure 10 [35]. Two units of square planar building blocks Co(II) 2,3,9,10,16,17,23,24-octahydroxyphthalocyaninato [(OH)_8_PcCo] were placed perpendicular to each other by the spiroborate linkers. The presence of minute cavities in the skeleton imparted rigidity to the structure. Moreover, the interpenetration was hindered by the short linker. The topologies with their expanded form are shown in Table 1. 

### 4.6. 3D Cage-COFs

**Figure 11:**
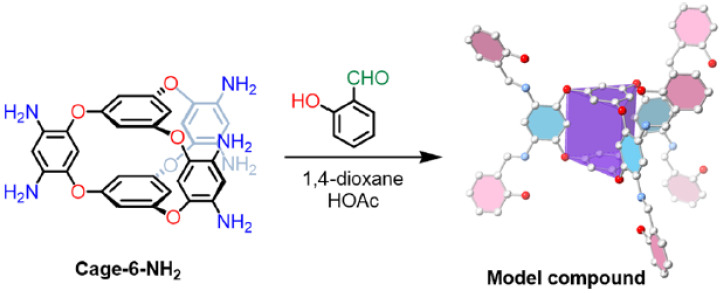
Synthesis of 3D Cage-COF [36].

Cage-6-NH_2_, a building block with the shape of a triangular prism, was used to design a 3D Cage-COF using 2,5-dihydroxyterephthalaldehyde (DHTPA) as the linear linker [36]. The 3D Cage-COF has an **acs** net topology, as shown Figure 11. 

Due to the absence of boronate ester or imine bonds, the Cage-6-NH_2_ moiety can form different COFs by linking with a variety of linkers. The trigonal prism can be extended along the three dimensions. The hydroxyl groups in 2,5-dihydroxyterephthalaldehyde form intramolecular hydrogen bonds with imine bonds and propel the formation of the cage structure. The interpenetration is limited to two-fold due to the presence of the bulky Cage-6-NH_2_ molecule. The synthesized Cage-COF showed a good uptake of carbon dioxide. The addition of dimethylformamide converted the topology of the 3D Cage-COF to a large-pore (lp), two-fold structure. 

An organic cage, 6NH_2_-OC.4HCl, was used for the synthesis of a 3D Cage-COF with triethylamine (TEA) as a linker [37]. The organic cage has a symmetric *D_3h_* conformation and single bonds in the backbone. The catalysts played a vital role in the formation of a highly crystalline structure. The acid catalysts failed to generate good crystals while the base catalysts were effective. The crystal has a two-fold interpenetrated **acs** topology. Although different structures are formed by the torsion and rotation of the flexible single bond present in the cage moiety, the structure becomes rigid after activation. The tuned conformations of the 3D Cage-COF formed two different contracted forms (3D Cage-COF-Cl and 3D Cage-COF-OH) and another expanded form (3D Cage-COF-H). The 3D Cage-COF-OH structure showed a good selective uptake of carbon dioxide from a mixture of carbon dioxide and methane due to the existence of abundant pores. The introduction of the chlorine or hydroxyl groups as substituents created a contracted structure due to the high noncovalent bonds between the two interpenetrated structures. 

## 5. Comparison of the 3D Crystals with Different Topologies

A comparative study was conducted among the 3D COF crystals reported within the last 6 years, as shown in Table 2. Among all the reported crystals, the Brunauer–Emmett–Teller (BET) surface area is the highest (5083 m^2^g^−1^) for Ni-DBA-3D-COF with a **bor (P4̅3*m*)** net topology [30]. A Ni(0) complex is formed where Ni binds to the cavity of dehydrobenzoannulene and there is no link at the boronate ester bonds in the crystal. This topology has favored the formation of 3D crystals with maximum surface area. The presence of dehydrobenzoannulene as soft electron-donating ligands and its coordination with the metal play a vital role in the formation of the crystal with a large surface area and low density (0.13 g cm^−3^). The next highest BET surface area (3383 m^2^g^−1^) is observed for a 3D triptycene-based COF having an **stp** topology [19]. 

The difference between the surface areas of Ni-DBA-3D-COF and the triptycene-based COF is considerably large. The crystal showed two different pore sizes of 41 Å along the *c*-axis and 15 Å along both the *a-* and *b*-axes. Moreover, the crystal is also light, with a density of 0.108 g cm^−3^. The large pores absorbed myoglobin easily. The combination of [6 + 4] in 2D-*D_2h_* (a pyrene-based monomer) and 3D-*D_3h_* (a triptycene-based building block) formed the 3D COF-triptycene. 

JUC 552 is a 3D meso-COF with the next highest surface area (3023 m^2^ g^−1^) with a non-interpenetrated **dia** topology. However, the size of the pores (26.5 Å) is comparatively smaller. These COFs were modulated by altering the methoxy substituents of the tetrahedral building blocks based on the concept of steric hindrance. A highly substituted linker imparted structural rigidity and reduced the dynamic nature of the skeleton. In a similar line, the surface area of 3D COF-PI-1 having polyimide as a monomer has a surface area of 2403 m^2^ g^−1^. The crystal is created with a combination of both non-interpenetrated and four-fold penetrated **dia** topology. Another 3D COF, 3D-COF-FPBA, with a **ctn** topology also has a noticeably high surface area and large pore size (13.6 Å). 

This study revealed that 3D SiCOF-5 with **srs-c** net has the lowest surface area among all the COFs. Thus, the silica-based COF with **srs** nets has a smaller surface area compared to that of the other 3D COFs. The 3D Cage-COFs have an **acs** topology with considerable surface areas and pore sizes. It is noteworthy to mention that the metalated 3D-COFs exhibit slightly different properties than their nonmetalated counterparts. 

## 6. Application of 3D COFs

### 6.1. Photocatalysts

**Scheme 2:**
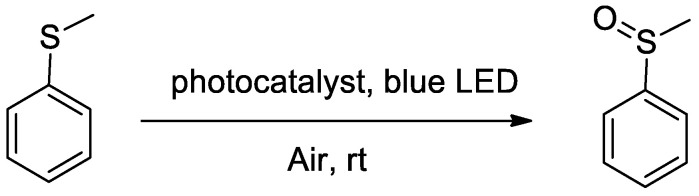
Photocatalytic oxidation of aromatic sulphur compounds.

The large surface and abundant pores are two remarkable characteristics of 3D COFs. Nevertheless, the application of 3D COFs is restricted due to a narrow range of building blocks and topologies. Porphyrin-based 3D COFs are good photocatalysts for producing oxygen in its singlet state [24]. Moreover, metalated (Cu and Pd) porphyrin-based 3D COFs showed better photocatalytic activities [49]. The 3D porphyrin-based Pd-COF photocatalyzed the oxidation of sulphides to sulphoxides. The excited triplet state is feasibly quenched by the stacked blocks of palladium porphyrin. Another Pd-porphyrin-based 3D COF photocatalyzed the oxidation of thioanisole to methyl phenyl sulfoxide with 98% yield, as shown in Scheme 2 [49]. The catalyst remained active after three repetitions. A superoxide radical anion was formed by the transfer of electrons from triplet porphyrin to molecular oxygen. 

### 6.2. Drug Delivery

**Figure 12:**
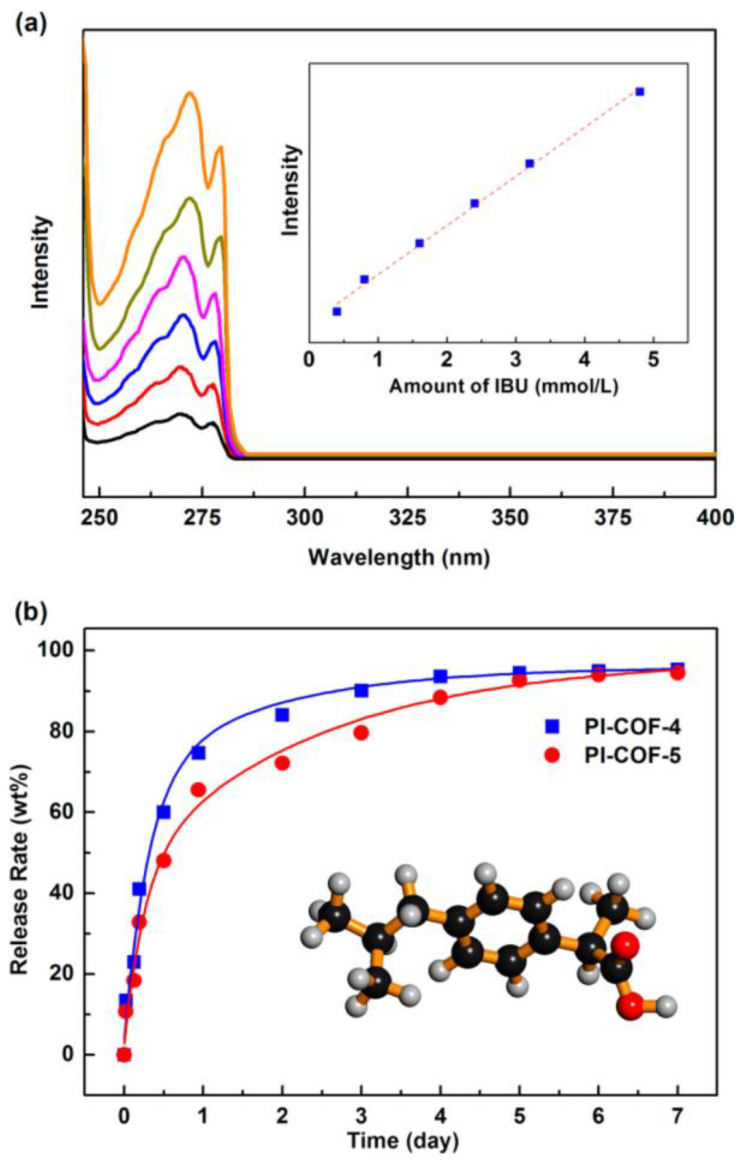
(**a**,**b**) PI-COFs with high surface areas for drug delivery. Reprinted (adapted) with permission from [47]. Copyright {2015} American Chemical Society.

Ibuprofen, captopril, and caffeine were selected as the model drugs for studying the controlled in vitro delivery of drugs by a polyimide-based 3D COF. The large pores and stability of the COF were responsible for the efficient entrapment and release of the drug molecule [47], as shown in Figure 12. 

### 6.3. Fluorescence

A pyrene-based 3D COF demonstrated yellow-green fluorescent properties and has the potential for the identification of picric acid [32]. The fluorescence is quenched in the presence of picric acid. The porous 3D COFs have the potential to selectively separate carbon dioxide gas from a mixture of carbon dioxide and nitrogen. An aggregation-induced emission-based 3D COF exhibited a fluorescence property by emitting yellow light of 543 nm and 20% photoluminescence quantum yield (PLQY) [50]. This phenomenon is attributed to the aggregation of tetraphenylethylene blocks within the 3D skeleton. The relaxation of the tetraphenylethylene blocks is retarded by the rigid skeleton, leading to an increase in the photoluminescence quantum yield. The fluorescence was quenched in the presence of picric acid.

**Figure 13:**
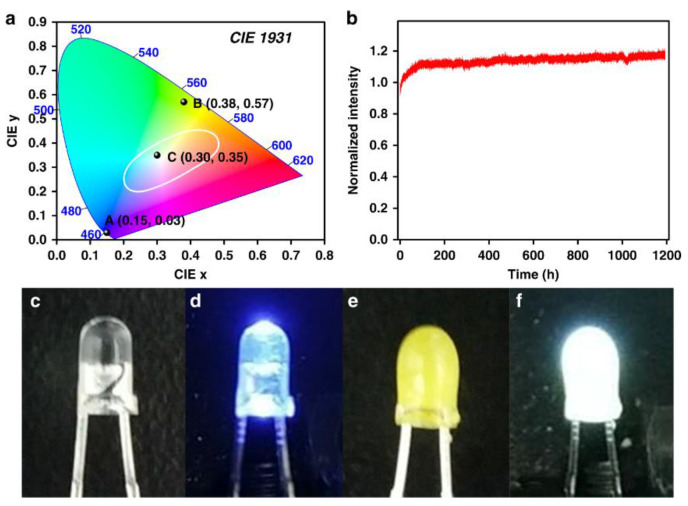
(**a**–**f**) A 3D-TPE-COF for white LED [50].

A white light-emitting diode (WLED) was fabricated by coating a fluorescent aggregation-induced emission-based 3D COF on a blue LED bulb for emitting white light, which paved the way for white light-emitting diodes devoid of rare-earth metals, as displayed in Figure 13 [50].

### 6.4. Catalysts

**Scheme 3:**
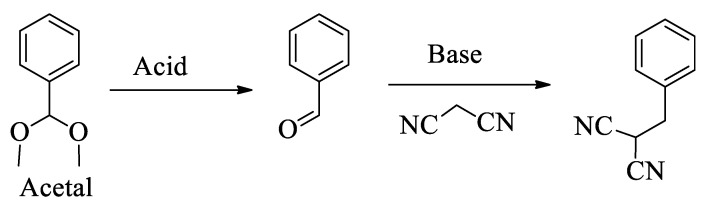
Conversion of acetal catalyzed by the synthesized COF.

A 3D COF having two different linkers, boroxine and imine, showed good bifunctional catalytic properties [39]. The imine groups present in the linkers are basic sites while the boroxine groups are the acidic sites. The catalyst hydrolyzed an acetal using the boroxine group and Knoevenagel condensation was later catalyzed by the basic imine linkage, as shown in Scheme 3. 

**Scheme 4:**
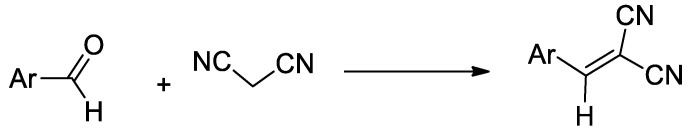
A 3D COF catalyzed Knoevenagel condensation reaction.

Another pyridyl-based 3D COF was used as a heterogeneous catalyst for another Knoevenagel condensation reaction, as shown in Scheme 4 [51]. The pyridyl groups are the Lewis base for catalyzing the reaction between malanonitrile and an aromatic aldehyde with a yield of 72%. 

An Ni-dehydrobenzoannulene-based 3D COF can be used to catalyze the polymerization of ethylene [30]. 

**Scheme 5:**
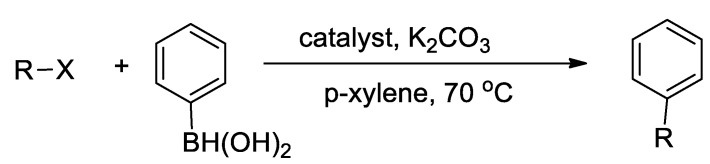
Suzuki–Miyaura reaction catalyzed by 3D COF.

A Pd(II)-spirobifluorene-based 3D COF catalyzed the Suzuki–Miyaura reaction between phenylboronic acid and aryl halide, as shown in Scheme 5 [52]. The reaction furnished products with a 97% yield. The high catalytic efficiency can be attributed to the fact that the agglomeration of Pd(0) was reduced by the metal-binding bipyridine sites. These sites were well-organized and were present on the COF skeleton and resembled ligands. Thus, a large number of available active sites were present on the crystal. 

An Ni-dehydrobenzoannulene-based 3D COF effectively separated ethane and ethylene from a mixture [30]. The uptake capacity of both the gases was higher by the metalated 3D COF as compared to that of the nonmetalated counterpart. This indicates that metal plays a pivotal role in the separation of gases. There are two factors responsible for the increased uptake of the gases by Ni containing 3D COF: (1) a weak complex is formed by the interaction between the ethylene π orbitals and Ni(0) d-orbitals [32]; (2) ethane is polarized by the open Ni(0) sites [53]. 

**Figure 14:**
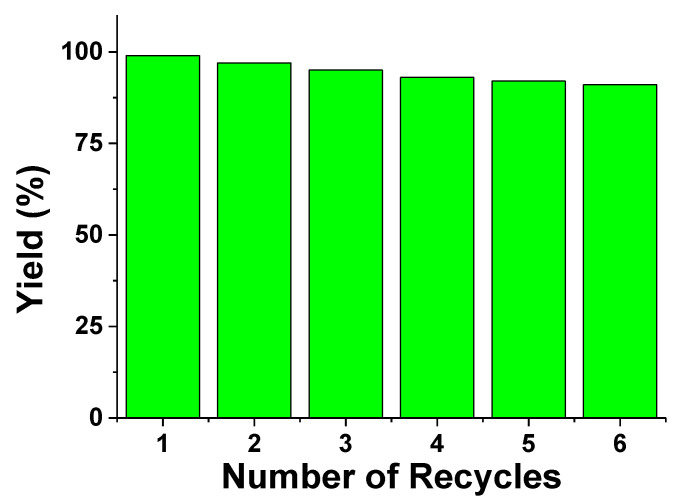
Recycle test of 3D COF (3D-HNU5) for CO_2_ capture.

An azine-linked 3D COF was prepared with high porosity and excellent thermal stability, which illustrated good performance for CO_2_ capture under mild conditions after six runs (yield > 90% and perfect turnover number), as shown in Figure 14 [54]. This heterogeneous catalyst is highly active due to its large specific area and intrinsic porous structure.

**Figure 15:**
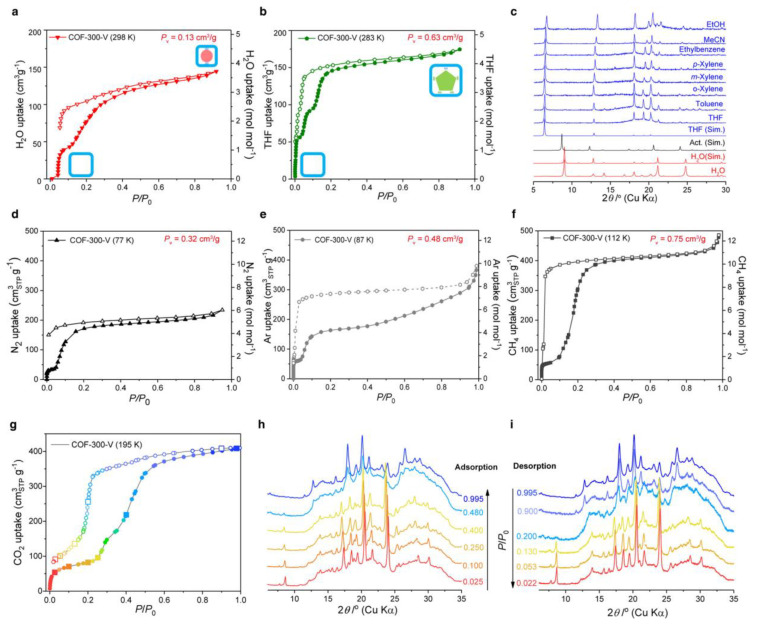
(**a–i**) Guest-dependent dynamics using iconic COF-300 with high crystallinity Reprinted (adapted) with permission from [55]. Copyright {2019} American Chemical Society.

Another imine-based 3D COF demonstrated the uptake of tetrahydrofuran, cyclohexane, and 1,4-dioxane as the adsorption characteristics were dependent on the guest molecule, as shown in Figure 15 [55]. This switching of the structure can have future applications as probes for detection [56,57]. Carbon dioxide also imparted exchangeability to the molecule. 

### 6.5. Absorption

1,4-phthalaldehyde-derived 3D COF is highly porous and exhibited an iodine uptake capacity of 82.4 wt% at room temperature [41]. A charge-transfer complex is formed between iodine and the conjugated system formed by the integration of imine linkers and phenyl rings. A high amount of iodine uptake is facilitated in the 3D COF pores due to the charge-transfer complex. The conductivity of the iodine-absorbed 3D COF is augmented due to a transfer of charge between the π-conjugated walls of the channels and iodine. Additionally, the one-dimensional channels accommodated the iodine molecules. Although the crystal is soft and undergoes local deformation, it can be used for iodine absorption repeatedly with an 80% efficacy. Furthermore, 3D ionic COFs synthesized using ethidium bromide or diimidium bromide were used for the abstraction of permanganate ions resembling technetium (Tc-99), a toxic ion present in nuclear wastes [47]. The ion-exchange capability was reversible, fast, and repeatable. Moreover, the COF selectively encapsulated methyl orange dye from a mixture of dyes comprising methyl orange and methylene blue. 

An enantiopure tera-aldehyde-based chiral 3D COF was used as the packing material of columns and was used for the resolution of stereoisomers, 1-phenyl-2-propanol with a good chromatographic resolution (*R*_s_ = 1.52) [58,59,60]. The *S*-enantiomer was obtained initially, followed by the *R*-enantiomer. A combination of channels resembling chiral tubes and amphiphilic tubes was present in the 3D COF. The inner part of the channels was covered by a layer of chiral hydroxyl groups. These chiral hydroxyl groups interacted with the guest alcohol groups and caused selective adsorption. The COF-based column could not separate a racemic mixture indicating, its potential for the identification of chirality.

**Figure 16:**
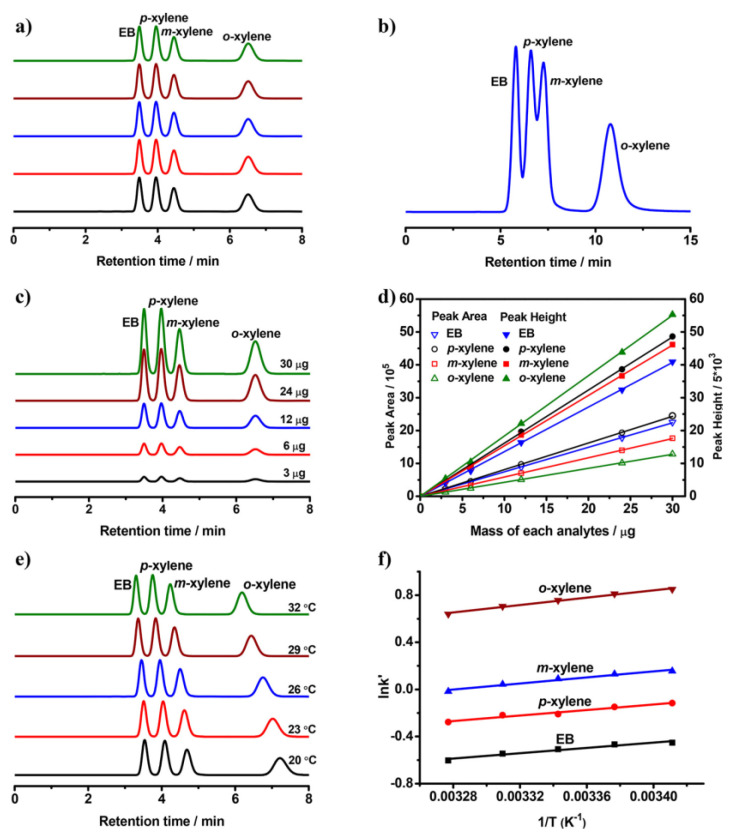
Salen-COFs for separation of C_8_ alkyl-aromatics. Reprinted (adapted) with permission from [29]. Copyright {2019} American Chemical Society. (**a**) HPLC chromatograms of EB and xylene isomers on the COF 1 packed column for five replicate experiments; (**b**) HPLC chromatograms of EB and xylene isomers on the COF 1-Zn packed column; (**c**) HPLC chromatograms of EB and xylene isomers with different injected masses on the COF 1 packed column; (**d**) Effects of injected mass of EB and xylene isomers on the peak area and the peak height; (**e**) HPLC chromatograms of EB and xylene isomers on the COF 1 packed column at 20–32 °C; (**f**) Van’t Hoff plots for EB and xylene isomers. Analytes were analyzed at r t. using hexane and DCM (*v*/*v* = 95:5) at a flow rate of 0.5 mL min^−1^ with UV detection at 254 nm.

Another salen-based 3D COF was used as a stationary phase in high-pressure liquid chromatography (HPLC) columns for the efficient separation of ethylbenzene and xylene isomers, as shown in Figure 16 [29].

### 6.6. Dopants

The conjugation in spirobifluorene-based COFs was used for harvesting solar energy by doping photoelectric devices with the COFs [61]. Spirobifluorene, with a biplanar structure, is the tetrahedral node having an ordered porous structure. Multiple channels for transporting electrons are present within the interpenetrated and highly ordered geometry. The doping of the COF into the CH_3_NH_3_PbI_3_ film reduced the leakage and showed good diode properties.

**Figure 17:**
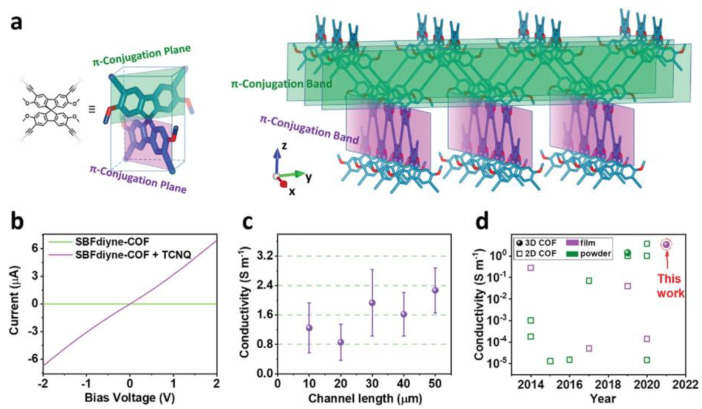
(**a**–**d**) Schematic illustrating of conductive SBFdiyne-COF film [63].

SBFdiyne-based 3D COF films doped with tetracyanoquinodimethane (TCNQ) showed a high conductivity due to the existence of orthogonally placed conjugated polymers in the COF [62,63], as shown in Figure 17. These conjugated polymers are channels for transporting the charges. A charge-transfer complex is effectively formed due to the electron-donating methoxy groups [64]. The uptake of doped material is high as the film is highly porous. Boundaries and defects are absent in the films. Hence, these films have the potential to be used as electrodes.

### 6.7. Radical Quenching

**Figure 18:**
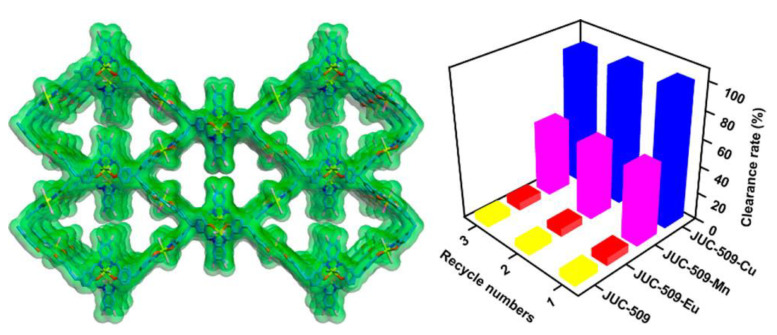
The 3D salphen-based COFs as catalytic antioxidants. Reprinted (adapted) with permission from [27]. Copyright {2019} American Chemical Society.

Cu(II) containing 3D salphen-based COFs eliminated superoxide radical anion (O_2_^•−^) [27], which is used in biology and medicine (Figure 18). The dismutation reaction of O_2_^•−^ is catalyzed by redox-active metal centers in salens. The chemical stability, biocompatibility, and large surface area are effective at removing the oxygen radical. The redox-active metal center is responsible for the catalytic activity. However, a high concentration of the catalyst is required for the removal of oxygen radicals.

## 7. Conclusions

The review summarizes the synthesis and potential application of different 3D COFs. The major limitations of 3D COFs are fewer building blocks and topologies. Nonplanar building blocks with suitable functional groups are still limited to be applied as nodes in the synthesis of 3D COFs. Since designing a topology is the basic criterion for creating new 3D COFs, new building units should be further investigated.

The functionalization of 3D COFs is significant for different applications. Furthermore, 3D COFs are functionalized at four different points: groups introduced during the latter part of synthesis, building blocks, linkages, and guest molecules. The groups introduced later can destroy the crystal structure. However, crystallization problems can be thwarted by introducing functional groups at the later part of the synthesis process. Building blocks and linkages are uniformly distributed throughout the framework. However, there are limited suitable linkages and functional groups as inappropriate functional groups tend to destroy the crystallinity of 3D COFs.

The recyclability and stability of guest molecules are low. Thus, the functionalization of 3D COFs requires an extensive study to synthesize new 3D COFs using new functional groups. Additionally, both 2D and 3D COFs can be prepared utilizing the same functional groups and their diverse properties can be explored.

Although 3D COFs have been explored as heterogeneous catalysts, the storing of gas and their separation, chemical sensing, fluorescence, and absorbing materials, there is a huge scope for their applications in diverse areas that are yet to be investigated. Design methods are required to obtain non-interpenetrated structures having large pore sizes and surface areas for further applications.

## Figures and Tables

**Table 1:** Topologies with their abbreviated form.

Topology	Abbreviation
Boracite net	**bor**
Diamondoid net	**dia**
Quartz net	**qtz**
The net formed by Pt and S atoms	**pts**
Body-centered cubic net	**bcu**
Primitive cubic	**pcu**

**Table 2:** The parameters and topology of different 3D COFs.

Type of 3D COFs	Topology	Pore Size	BET Surface Area m^2^ g^−1^	Pore Volume cm^3^ g^−1^	Ref.
Ni-DBA-3D-COF	bor	2.6 nm	503	1.59	[30]
3D-Py-COF	pts		1290	0.72	[38]
3D-COF-FPBA	ctn	13.6 Å	2259		[39]
3D-COF-FFPBA	ctn	12.8	2071		
CD-COF-Li	rra		760	0.39	[40]
COF-DL229	dia	1.4 nm	1762	0.64	[41]
3D SiCOF-5	srs		370		[42]
JUC 550 No Me	dia	10.6 Å	846		[43]
JUC 551 2Me	dia	14.0 Å, 22.0 Å	1728		
JUC 552 4 Me	dia	26.5 Å	3023		
3D COF-triptycene	stp	15 Å, 41 Å	3383		[19]
3D COF-BMTA	pts	0.85 nm	1650		[44]
3D COF-HBC	pcu	0.63 nm	778		[45]
3D COF-triptycene	ceq	10 Å, 16 Å	1148		[22]
3D COF-TPB-Ph	ljh	1 nm	1430		[46]
3DCOF-TPB-OMe	pts	0.52 nm	880		
3D COF-SB	nbo	2.45 nm	1726	0.9	[35]
3D COF-PI-1	dia	13 Å	2403		[47]
3D COF-PI-2	dia	10 Å	1876		
3D COF-ionic-DB	dia	8.6 Å	966		[48]
3D COF-ionic-EB	dia	8.2 Å	880		

## Data Availability

MDPI Research Data Policies.

## References

[1] Vardhan H., Nafady A., Al-Enizi A.M., Ma S. (2019). Pore surface engineering of covalent organic frameworks: Structural diversity and applications. Nanoscale.

[2] Guan X., Chen F., Fang Q., Qiu S. (2020). Design and applications of three dimensional covalent organic frameworks. Chem. Soc. Rev..

[3] Ta Q.T.H., Cho E., Sreedhar A., Noh J.-S. (2019). Mixed-dimensional, three-level hierarchical nanostructures of silver and zinc oxide for fast photocatalytic degradation of multiple dyes. J. Catal..

[4] Ma X., Scott T.F. (2018). Approaches and challenges in the synthesis of three-dimensional covalent-organic frameworks. Commun. Chem..

[5] Karak S., Dey K., Torris A., Halder A., Bera S., Kanheerampockil F., Banerjee R. (2019). Inducing Disorder in Order: Hierarchically Porous Covalent Organic Framework Nanostructures for Rapid Removal of Persistent Organic Pollutants. J. Am. Chem. Soc..

[6] Dey K., Pal M., Rout K.C., Kunjattu H.S., Das A., Mukherjee R., Kharul U.K., Banerjee R. (2017). Selective Molecular Separation by Interfacially Crystallized Covalent Organic Framework Thin Films. J. Am. Chem. Soc..

[7] Dey K., Kunjattu H.S., Chahande A.M., Banerjee R. (2020). Nanoparticle Size-Fractionation through Self-Standing Porous Covalent Organic Framework Films. Angew. Chem. Int. Ed..

[8] Kandambeth S., Dey K., Banerjee R. (2019). Covalent Organic Frameworks: Chemistry beyond the Structure. J. Am. Chem. Soc..

[9] Gui B., Lin G., Ding H., Gao C., Mal A., Wang C. (2020). Three-Dimensional Covalent Organic Frameworks: From Topology Design to Applications. Acc. Chem. Res..

[10] Huang N., Wang P., Jiang D. (2016). Covalent organic frameworks: A materials platform for structural and functional designs. Nat. Rev. Mater..

[11] Phan P.T., Hong J., Tran N., Le T.H. (2023). The Properties of Microwave-Assisted Synthesis of Metal–Organic Frameworks and Their Applications. Nanomaterials.

[12] An S., Xu Q., Ni Z., Hu J., Peng C., Zhai L., Guo Y., Liu H. (2021). Construction of Covalent Organic Frameworks with Crown Ether Struts. Angew. Chem. Int. Ed..

[13] Xie Y., Li J., Lin C., Gui B., Ji C., Yuan D., Sun J., Wang C. (2021). Tuning the Topology of Three-Dimensional Covalent Organic Frameworks via Steric Control: From pts to Unprecedented ljh. J. Am. Chem. Soc..

[14] Zhao C., Diercks C.S., Zhu C., Hanikel N., Pei X., Yaghi O.M. (2018). Urea-Linked Covalent Organic Frameworks. J. Am. Chem. Soc..

[15] Horike S., Shimomura S., Kitagawa S. (2009). Soft porous crystals. Nat. Chem..

[16] Ta Q.T.H., Tran N.M., Tri N.N., Sreedhar A., Noh J.-S. (2021). Highly Surface-Active Si-Doped TiO2/Ti3C2Tx Heterostructure for Gas Sensing and Photodegradation of Toxic Matters. Chem. Eng. J..

[17] Geng K., He T., Liu R., Dalapati S., Tan K.T., Li Z., Tao S., Gong Y., Jiang Q., Jiang D. (2020). Covalent Organic Frameworks: Design, Synthesis, and Functions. Chem. Rev..

[18] Ta Q.T.H., Thakur D., Noh J.-S. (2022). Enhanced Gas Sensing Performance of ZnO/Ti3C2Tx MXene Nanocomposite. Micromachines.

[19] Li H., Ding J., Guan X., Chen F., Li C., Zhu L., Xue M., Yuan D., Valtchev V., Yan Y. (2020). Three-Dimensional Large-Pore Covalent Organic Framework with stp Topology. J. Am. Chem. Soc..

[20] Wang Y., Wu C., Sun W., Pan Q., Hao W., Liu H., Sun J., Li Z., Sun J., Zhao Y. (2021). Triptycene-based three-dimensional covalent organic frameworks with stp topology of honeycomb structure. Mater. Chem. Front..

[21] Kang X., Han X., Yuan C., Cheng C., Liu Y., Cui Y. (2020). Reticular Synthesis of tbo Topology Covalent Organic Frameworks. J. Am. Chem. Soc..

[22] Li Z., Sheng L., Wang H., Wang X., Li M., Xu Y., Cui H., Zhang H., Liang H., Xu H. (2021). Three-Dimensional Covalent Organic Framework with ceq Topology. J. Am. Chem. Soc..

[23] Martínez-Abadía M., Strutyński K., Lerma-Berlanga B., Stoppiello C.T., Khlobystov A.N., Martí-Gastaldo C., Saeki A., Melle-Franco M., Mateo-Alonso A. (2021). π-Interpenetrated 3D Covalent Organic Frameworks from Distorted Polycyclic Aromatic Hydrocarbons. Angew. Chem. Int. Ed..

[24] Lin G., Ding H., Chen R., Peng Z., Wang B., Wang C. (2017). 3D Porphyrin-Based Covalent Organic Frameworks. J. Am. Chem. Soc..

[25] Liu Y., Ma Y., Zhao Y., Sun X., Gándara F., Furukawa H., Liu Z., Zhu H., Zhu C., Suenaga K. (2016). Weaving of organic threads into a crystalline covalent organic framework. Science.

[26] Miller M.T., Gantzel P.K., Karpishin T.B. (1998). Structures of the Copper(I) and Copper(II) Complexes of 2,9-Diphenyl-1,10-phenanthroline:  Implications for Excited-State Structural Distortion. Inorg. Chem..

[27] Yan S., Guan X., Li H., Li D., Xue M., Yan Y., Valtchev V., Qiu S., Fang Q. (2019). Three-dimensional Salphen-based Covalent–Organic Frameworks as Catalytic Antioxidants. J. Am. Chem. Soc..

[28] Zhang X.M., Tang J., Wang L.N., Yao D., Yu Q., Huang F.P., Bian H.D. (2015). Superoxide dismutase activity studies of Mn(III)/Co(III)/Fe(III) complexes with Schiff base ligands. Polyhedron.

[29] Huang J., Han X., Yang S., Cao Y., Yuan C., Liu Y., Wang J.-G., Cui Y. (2019). Microporous 3D Covalent Organic Frameworks for Liquid Chromatographic Separation of Xylene Isomers and Ethylbenzene. J. Am. Chem. Soc..

[30] Baldwin L.A., Crowe J.W., Pyles D.A., McGrier P.L. (2016). Metalation of a Mesoporous Three-Dimensional Covalent Organic Framework. J. Am. Chem. Soc..

[31] Ferrara J.D., Tessier-Youngs C., Youngs W.J. (1985). Synthesis and characterization of the first transition metal complex of 1,2:5,6:9,10-tribenzocyclododeca-1,5,9-triene-3,7,11-triyne. J. Am. Chem. Soc..

[32] Safarik D.J., Eldridge R.B. (1998). Olefin/Paraffin Separations by Reactive Absorption:  A Review. Ind. Eng. Chem. Res..

[33] Liang W., Xu F., Zhou X., Xiao J., Xia Q., Li Y., Li Z. (2016). Ethane selective adsorbent Ni(bdc)(ted)0.5 with high uptake and its significance in adsorption separation of ethane and ethylene. Chem. Eng. Sci..

[34] Lu H., Han W., Yan X., Chen C., Niu T., Gu Z. (2021). A 3D Anionic Metal Covalent Organic Framework with soc Topology Built from an Octahedral TiIV Complex for Photocatalytic Reactions. Angew. Chem. Int. Ed..

[35] Wang X., Bahri M., Fu Z., Little M.A., Liu L., Niu H., Browning N.D., Chong S.Y., Chen L., Ward J.W. (2021). A Cubic 3D Covalent Organic Framework with nbo Topology. J. Am. Chem. Soc..

[36] Zhu Q., Wang X., Clowes R., Cui P., Chen L., Little M.A., Cooper A.I. (2020). 3D Cage COFs: A Dynamic Three-Dimensional Covalent Organic Framework with High-Connectivity Organic Cage Nodes. J. Am. Chem. Soc..

[37] Ji C., Su K., Wang W., Chang J., El-Sayed E.-S.M., Zhang L., Yuan D. (2022). Tunable Cage-Based Three-Dimensional Covalent Organic Frameworks. CCS Chem..

[38] Lin G., Ding H., Yuan D., Wang B., Wang C. (2016). A Pyrene-Based, Fluorescent Three-Dimensional Covalent Organic Framework. J. Am. Chem. Soc..

[39] Li H., Pan Q., Ma Y., Guan X., Xue M., Fang Q., Yan Y., Valtchev V., Qiu S. (2016). Three-Dimensional Covalent Organic Frameworks with Dual Linkages for Bifunctional Cascade Catalysis. J. Am. Chem. Soc..

[40] Zhang Y., Duan J., Ma D., Li P., Li S., Li H., Zhou J., Ma X., Feng X., Wang B. (2017). Three-Dimensional Anionic Cyclodextrin-Based Covalent Organic Frameworks. Angew. Chem. Int. Ed..

[41] Wang C., Wang Y., Ge R., Song X., Xing X., Jiang Q., Lu H., Hao C., Guo X., Gao Y. (2018). A 3D Covalent Organic Framework with Exceptionally High Iodine Capture Capability. Chem.-Eur. J..

[42] Yahiaoui O., Fitch A.N., Hoffmann F., Fröba M., Thomas A., Roeser J. (2018). 3D Anionic Silicate Covalent Organic Framework with srs Topology. J. Am. Chem. Soc..

[43] Wang Y., Liu Y., Li H., Guan X., Xue M., Yan Y., Valtchev V., Qiu S., Fang Q. (2020). Three-Dimensional Mesoporous Covalent Organic Frameworks through Steric Hindrance Engineering. J. Am. Chem. Soc..

[44] Gao C., Li J., Yin S., Sun J., Wang C. (2020). Twist Building Blocks from Planar to Tetrahedral for the Synthesis of Covalent Organic Frameworks. J. Am. Chem. Soc..

[45] Qun G., Zhou L.-L., Dong Y.-B. (2022). Metalated covalent organic frameworks: From synthetic strategies to diverse applications. Chem. Soc. Rev..

[46] Phan P.T., Ta Q.T.H., Nguyen P.K.T. (2023). Designed Synthesis of Three-Dimensional Covalent Organic Frameworks: A Mini Review. Polymers.

[47] Fang Q., Wang J., Gu S., Kaspar R.B., Zhuang Z., Zheng J., Guo H., Qiu S., Yan Y. (2015). 3D Porous Crystalline Polyimide Covalent Organic Frameworks for Drug Delivery. J. Am. Chem. Soc..

[48] Li Z., Li H., Guan X., Tang J., Yusran Y., Li Z., Xue M., Fang Q., Yan Y., Valtchev V. (2017). Three-Dimensional Ionic Covalent Organic Frameworks for Rapid, Reversible, and Selective Ion Exchange. J. Am. Chem. Soc..

[49] Meng Y., Luo Y., Shi J., Ding H., Lang X., Chen W., Zheng A., Sun J., Wang C. (2020). 2D and 3D Porphyrinic Covalent Organic Frameworks: The Influence of Dimensionality on Functionality. Angew. Chem. Int. Ed..

[50] Ding H., Li J., Xie G., Lin G., Chen R., Peng Z., Yang C., Wang B., Sun J., Wang C. (2018). An AIEgen-based 3D covalent organic framework for white light-emitting diodes. Nat. Commun..

[51] Wang Y., Zhang G., Gao M., Cai Y., Zhan C., Zhao Z., Zhang D., Tang B.Z. (2017). Introductory lecture: Recent research progress on aggregation-induced emission. Faraday Discuss..

[52] Liu Y., Wu C., Sun Q., Hu F., Pan Q., Sun J., Jin Y., Li Z., Zhang W., Zhao Y. (2021). Spirobifluorene-Based Three-Dimensional Covalent Organic Frameworks with Rigid Topological Channels as Efficient Heterogeneous Catalyst. CCS Chem..

[53] Jiang W., Luo W., Wang J., Zhang M., Zhu Y. (2016). Enhancement of catalytic activity and oxidative ability for graphitic carbon nitride. J. Photochem. Photobiol. C Photochem. Rev..

[54] Guan P., Qiu J., Zhao Y., Wang H., Li Z., Shi Y., Wang J. (2019). A novel crystalline azine-linked three-dimensional covalent organic framework for CO_2_ capture and conversion. Chem Commun..

[55] Chen Y., Shi Z.-L., Wei L., Zhou B., Tan J., Zhou H.-L., Zhang Y.-B. (2019). Guest-Dependent Dynamics in a 3D Covalent Organic Framework. J. Am. Chem. Soc..

[56] Ma Y.-X., Li Z.-J., Wei L., Ding S.-Y., Zhang Y.-B., Wang W. (2017). A Dynamic Three-Dimensional Covalent Organic Framework. J. Am. Chem. Soc..

[57] Carrington E.J., McAnally C.A., Fletcher A.J., Thompson S.P., Warren M., Brammer L. (2017). Solvent-switchable continuous-breathing behaviour in a diamondoid metal–Organic framework and its influence on CO_2_ versus CH_4_ selectivity. Nat. Chem..

[58] Pang J., Yuan S., Qin J., Liu C., Lollar C., Wu M., Yuan D., Zhou H.-C., Hong M. (2017). Control the Structure of Zr-Tetracarboxylate Frameworks through Steric Tuning. J. Am. Chem. Soc..

[59] Guan X., Ma Y., Li H., Yusran Y., Xue M., Fang Q., Yan Y., Valtchev V., Qiu S. (2018). Fast, Ambient Temperature and Pressure Ionothermal Synthesis of Three-Dimensional Covalent Organic Frameworks. J. Am. Chem. Soc..

[60] Han X., Huang J., Yuan C., Liu Y., Cui Y. (2018). 3D Covalent Organic Frameworks for High Performance Liquid Chromatographic Enantioseparation. J. Am. Chem. Soc..

[61] Wu C., Liu Y., Liu H., Duan C., Pan Q., Zhu J., Hu F., Ma X., Jiu T., Li Z. (2018). Highly Conjugated Three-Dimensional Covalent Organic Frameworks Based on Spirobifluorene for Perovskite Solar Cell Enhancement. J. Am. Chem. Soc..

[62] Liu Y., Diercks C.S., Ma Y., Lyu H., Zhu C., Alshmimri S.A., Alshihri S., Yaghi O.M. (2019). 3D Covalent Organic Frameworks of Interlocking 1D Square Ribbons. J. Am. Chem. Soc..

[63] Yang Y., Mallick S., Izquierdo-Ruiz F., Schäfer C., Xing X., Rahm M., Börjesson K. (2021). A Highly Conductive All-Carbon Linked 3D Covalent Organic Framework Film. Small.

[64] Liu X., Li J., Gui B., Lin G., Fu Q., Yin S., Liu X., Sun J., Wang C. (2021). A Crystalline Three-Dimensional Covalent Organic Framework with Flexible Building Blocks. J. Am. Chem. Soc..

